# Clinicopathological and prognostic significance of HMGA2 overexpression in gastric cancer: a meta-analysis

**DOI:** 10.18632/oncotarget.19001

**Published:** 2017-07-05

**Authors:** Jingyi Zhu, Hailong Wang, Shuangnian Xu, Yingxue Hao

**Affiliations:** ^1^ Department of General Surgery, Southwest Hospital of Third Military Medical University, Chongqing, China; ^2^ Department of Hematology, Southwest Hospital of Third Military Medical University, Chongqing, China

**Keywords:** gastric cancer, HMGA2, prognosis, meta-analysis

## Abstract

**Background:**

High mobility group protein A2 (HMGA2) overexpression has been reported to be closely related to tumor progression [1-4] and indicate significantly worse overall survival in gastric cancer [5-8]. However, a final consensus regarding this issue has not yet been reached. Thus, we conducted a meta-analysis to evaluate the association between HMGA2 expression and prognosis of gastric cancer patients.

**Methods:**

The Cochrane Library, Embase, PubMed, Web of Science and China Biology Medicine databases were searched to identify eligible literature published prior to September 2016. In the included studies, the level of HMGA2 amplification was evaluated by immunohistochemistry. We performed a meta-analysis, and pooled relative risk (RRs), hazard ratio (HRs), and 95% confidence intervals (CIs) were analyzed using Review Manager 5.3.

**Results:**

Six studies [5-7, 9-11] involving 712 gastric cancer patients were included and stratified by HMGA2 amplification magnitude. The results of the analysis indicated that higher HMGA2 levels were associated with several clinicopathological parameters and predicted poor prognosis in terms of overall survival (OS).

**Conclusions:**

The results of the present study indicate that higher HMGA2 levels were significantly associated with TNM stage, lymph node status, vascular invasion, and poor OS in patients with gastric cancer. In conclusion, HMGA2 may serve as a promising prognostic biomarker in gastric cancer.

## INTRODUCTION

According to Globacan (2012), gastric cancer (GC) was the fifth most common carcinoma worldwide; at that time, the overall case fatality rate in GC patients was 74.5% [12]. Despite advances in chemotherapy and surgery, the prognosis of GC patients remains poor [13]. Most GC patients have advanced stage disease or distant metastases at the time of diagnosis because it is quite difficult to endoscopically diagnose early GC due to the subtle changes in endoscopic findings [14]. Prognosis is usually assessed by TNM staging (tumor, lymph nodes and metastasis). However, this approach may be flawed, as prognosis often differs in patients at the same tumor stage [15]. Given this fact, it is necessary to identify a specific prognostic biomarker that can accurately identify patients with poor prognosis, allowing health care professionals to preemptively alter their treatment strategy.

High mobility group protein A2 (HMGA2) is a small nonhistone chromosomal protein with three AT-hooks that can bind to the minor grooves of AT-rich regions of DNA [16]. HGMA2 has no intrinsic transcriptional activity but can affect transcription by altering chromatin architecture [17, 18]. Together with the HMGA2 gene, the HMGA1 gene encodes the HMGA protein. HMGA1 and HMGA2 are located on chromosomes 6p21 and 12q13-15, respectively [19]. High levels of HMGA2 expression have been identified during embryogenesis, but expression of this gene decreases in normal adult tissues, implying that HMGA2 may play a critical role in cell proliferation and differentiation during embryogenesis [20, 21]. HMGA2 has been found to be frequently amplified or subjected to chromosomal rearrangement. Numerous studies have reported that aberrant overexpression of HMGA2 is associated with increased invasion, stemness and poor prognosis in cancer [22-37]. The presence of a relationship between HMGA overexpression and malignant phenotypes has been supported by findings indicating the development of chemoresistance, spreading of metastases, and overall poor survival in most affected cases [1-3].

Nevertheless, the results of studies evaluating the prognostic value of HMGA2 in GC patients have been inconsistent. Several studies have shown that high levels of HMGA2 expression are associated with survival in/ and the clinicopathologic features of GC patients, including TNM stage, deep of invasion, and lymph node metastasis [5-7, 9-11]. The results of different studies have varied. Therefore, it is necessary to identify eligible studies and perform a meta-analysis to evaluate the prognostic value of HGMA2 in GC patients.

## RESULTS

### Study selection and characteristics

**Figure 1:**
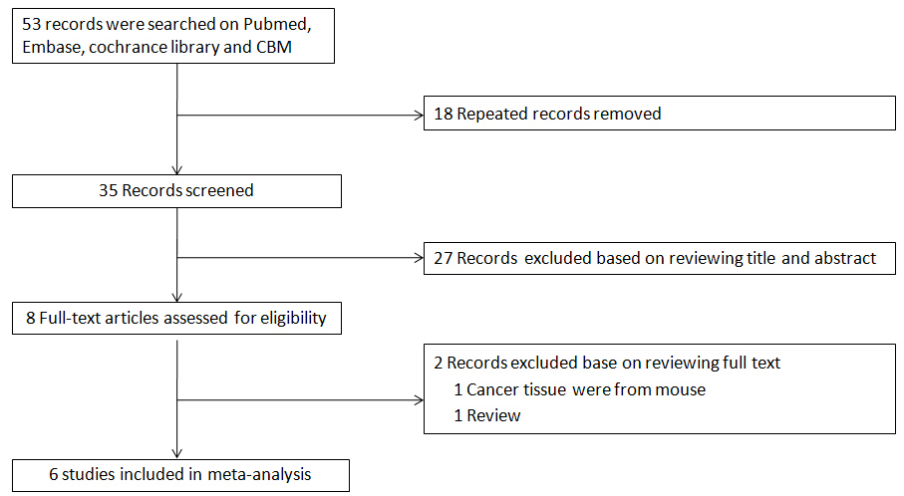
Flow chart of study selection

As shown in Figure 1, 53 studies were identified by searching the PubMed, Embase, Cochrane Library and Chinese Biology Medicine (CBM) databases. Finally, six eligible studies with 714 patients were included in the meta-analysis after the titles and abstracts of the articles were screened and full text articles were reviewed. The main characteristics of the included studies are displayed in tab. 1. The studies were confined to Asian countries, including China, Korea and Japan. A possible reason for the limited geographic distribution is that the incidence rates of GC have been found to be higher in eastern Asia (especially in China, Japan and Korea) than the rest of the world [39]. Of the six studies, four studies including a total of 531 patients provided OS data, and only one study including a total of 110 patients provided disease free survival data. Immunohistochemistry was the only method used to detect HMGA2 expression. It is noteworthy that none of the studies scored less than 6 on the NOS, suggesting that all included studies were of high methodological quality.

**Table 1:** Main characteristics of studies included in the meta-analysis

Study	Country	Number of patients	Sex	Median age	TNM stage	Deep of invasion	LN metastasis	Vascular invasion	Lymphatic invasion	Histological type	Size	Detection method	HMGA2-positive ratio(%)	Survival data type	Quanlity score
		HMGA2+vs HMGA2-	Male/ female		I+II/III+IV	T_1_+T_2_/ T_3_+T_4_	Present/ absent	Present/ absent	Present/ absent	Well+moderately/ poorly	>3cm/ <=3cm				
Dequan et al. (2014)	China	158 68vs90	104/54	NR	57/101	86/72	93/65	61/97	NR	117/41	63/95	IHC	43.0	OS	7
Junhy et al. (2015)	Korea	170 39vs131	123/47	61.5(29-89)	75/96	35/135	105/65	19/151	136/34	51/119	NR	IHC	22.9	OS	7
Kazuo et al. (2008)	Japan	110 55vs55	72/38	NR	59/51	67/43	78/32	30/80	78/32	55/55	87/23	IHC	50.0	OS	7
Kyong-Hwa et al. (2015)	Korea	110 72vs38	67/43	63.7(NR)	59/51	33/77	73/37	22/88	68/42	57/53	NR	IHC	65.5	DFS	7
LV Bonan et al. (2014)	China	93 68vs25	56/37	54(39-76)	26/67	33/60	55/38	NR	NR	NR	NR	IHC	73.1	OS	8
ZHA Lang et al. (2011)	China	71 46vs27	45/26	53(29-81)	25/46	NR	49/22	NR	NR	29/42	48/32	IHC	64.8	NR	6

Abbreviations: DFS=disease free survival, IHC = immunohistochemistry, LN= Lymph node, NA= not available, OS=overall survival.

### Expression and clinicopathological parameters

**Figure 2:**
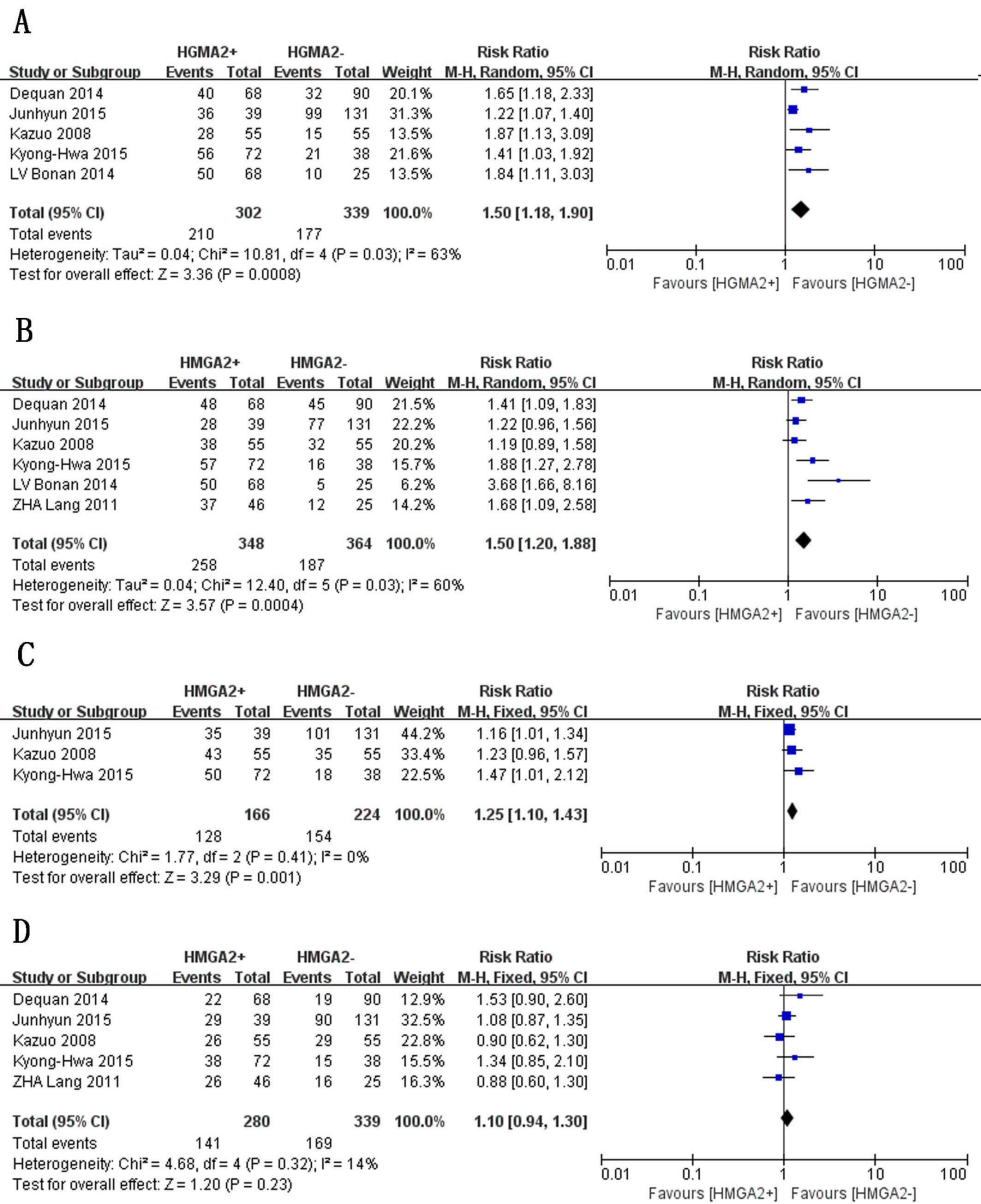
Forest plot of studies evaluating the associations between high HMGA2 expression and clinicopathological parameters **A**. Deep of invasion: T3+T4. **B**. Lymph node metastasis: present. **C**. Lymphatic invasion: present. **D**. Histological type: poorly differentiated.

**Figure 3:**
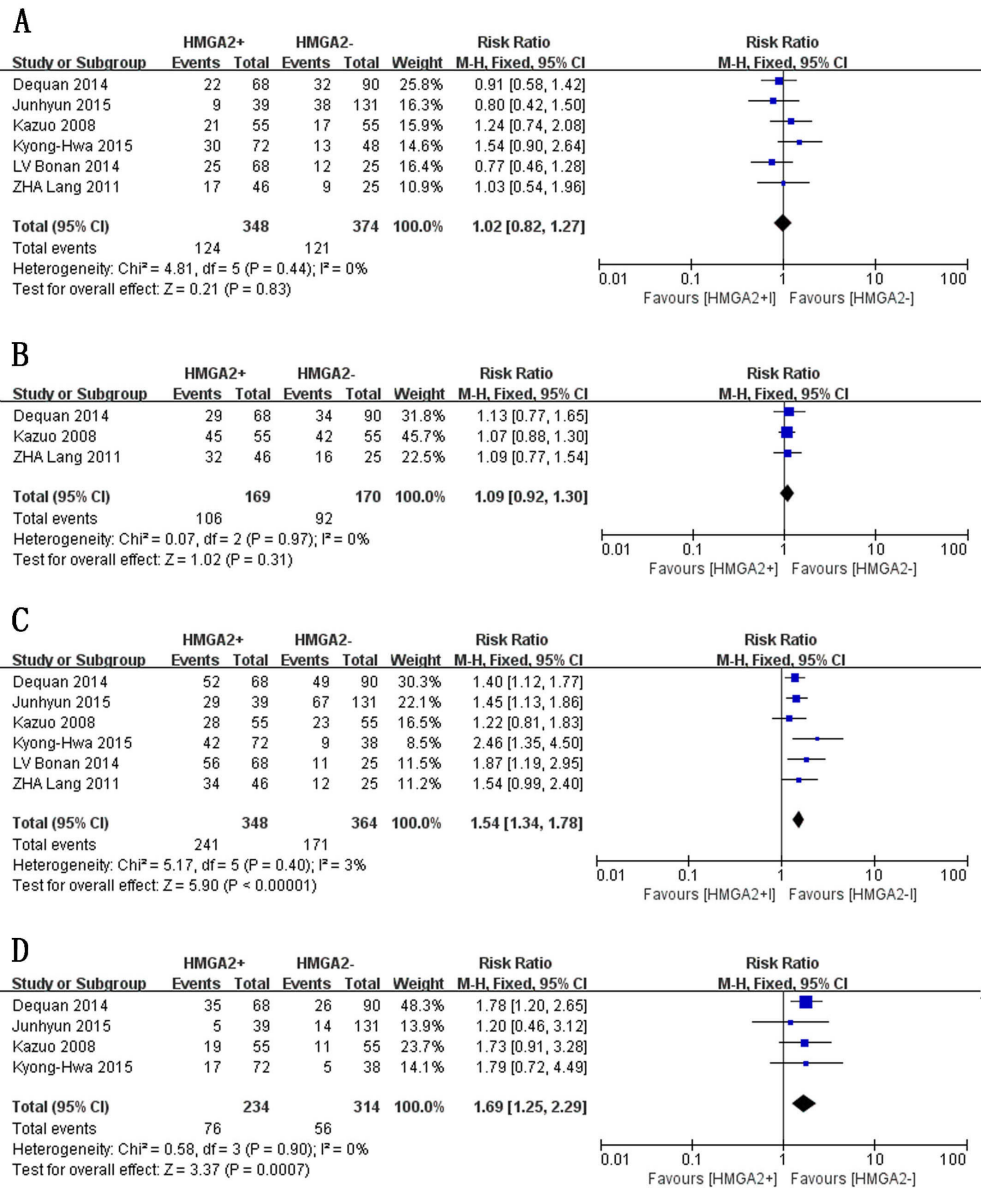
Forest plot of studies evaluating the associations between high HMGA2 expression and clinicopathological parameters **A**. Sex: female. **B**. Size: >3 cm. **C**. TNM stage: III+IV. **D**. Vascular invasion: present.

We assessed the associations between HMGA2 expression and clinicopathological parameters in GC patients. Since the I^2^ value for lymph node metastasis exceeded 50.0%, we used a random-effects model to pool these data. Otherwise, fixed-effects models were used. As illustrated in Figure 2 and Figure 3, the results of the meta-analysis indicated that increased HMGA2 expression was significantly associated with TNM stage (RR = 1.54, 95% CI = 1.34-1.78, *P* < 0.00001, fixed-effects model), T stage (RR = 1.50, 95% CI = 1.18-1.90, *P* =0.0008, random-effects model), vascular invasion (RR = 1.69, 95% CI = 1.25-2.29, *P* = 0.0007, fixed-effects model), lymph node metastasis (RR = 1.50, 95% CI = 1.20-1.88, *P* = 0.0004, random-effects model), and lymphatic invasion (RR =1.25, 95% CI = 1.10-1.43, *P* = 0.001, fixed-effects model). However, HMGA2 overexpression was not associated histological differentiation (RR =1.10, 95% CI = 0.94-1.30, *P* = 0.23, fixed-effects model), tumor size (RR =1.09, 95% CI = 0.92-1.30, *P* = 0.31, fixed-effects model) or sex (RR =1.02, 95% CI =0.82-1.27, *P* = 0.83, fixed-effects model). The stratified data indicated that the tumors of GC patients with high levels of HMGA2 expression tended to exhibit aggressive biological behavior.

### Expression and overall survival

**Figure 4:**
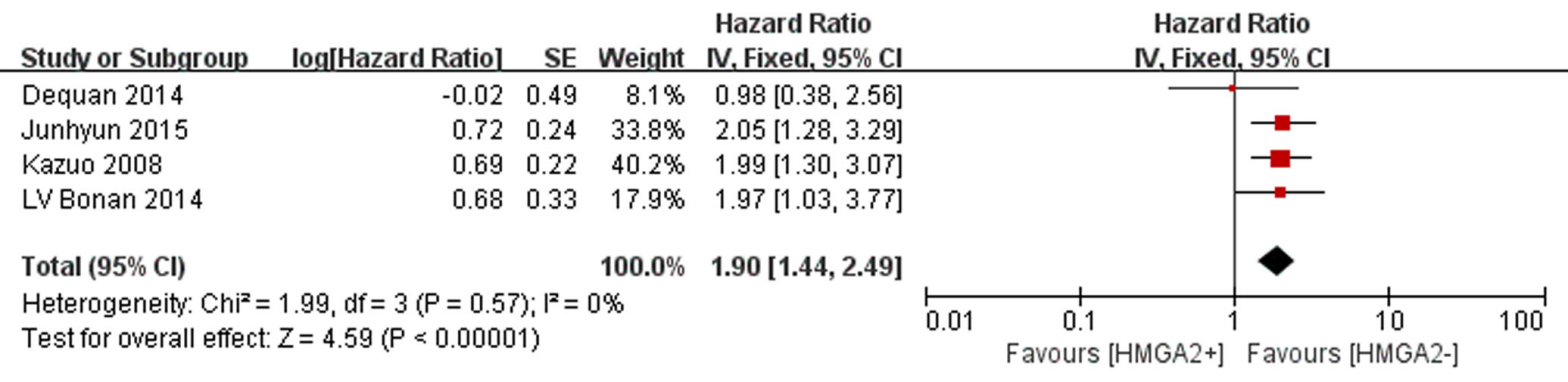
Forest plot of studies evaluating the association between high HMGA2 expression and OS

As shown in Figure 4, four studies evaluated OS. No obvious heterogeneity was identified across these studies (*P* = 0.57, I 2 = 0%); therefore, a fixed-effects model was used to calculate the pooled HR and 95% CI. As seen in Figure 4, the pooled data showed that elevated HMGA2 was significantly associated with poorer OS in GC (HR = 1.90 95% CI = 1.44-2.49, *P* < 0.00001).

### Publication bias and sensitivity analysis

**Figure 5:**
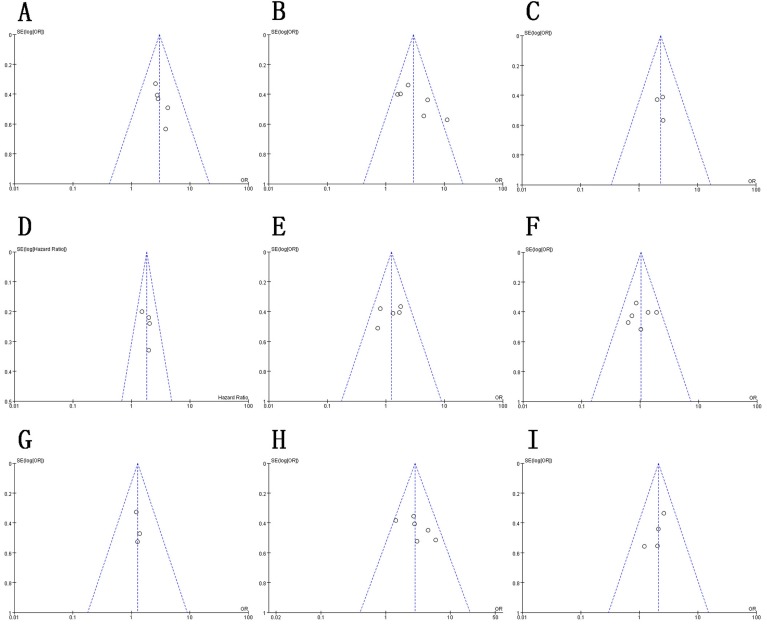
Funnel plot for publication bias in HMGA2-related studies **A.** Depth of invasion. **B**. Lymph node metastasis. **C**. Lymphatic invasion. **D.** OS. **E.** Histological type. **F**. Sex. **G.** Size. **H.** TNM stage. **I**. Vascular invasion..

**Figure 6:**
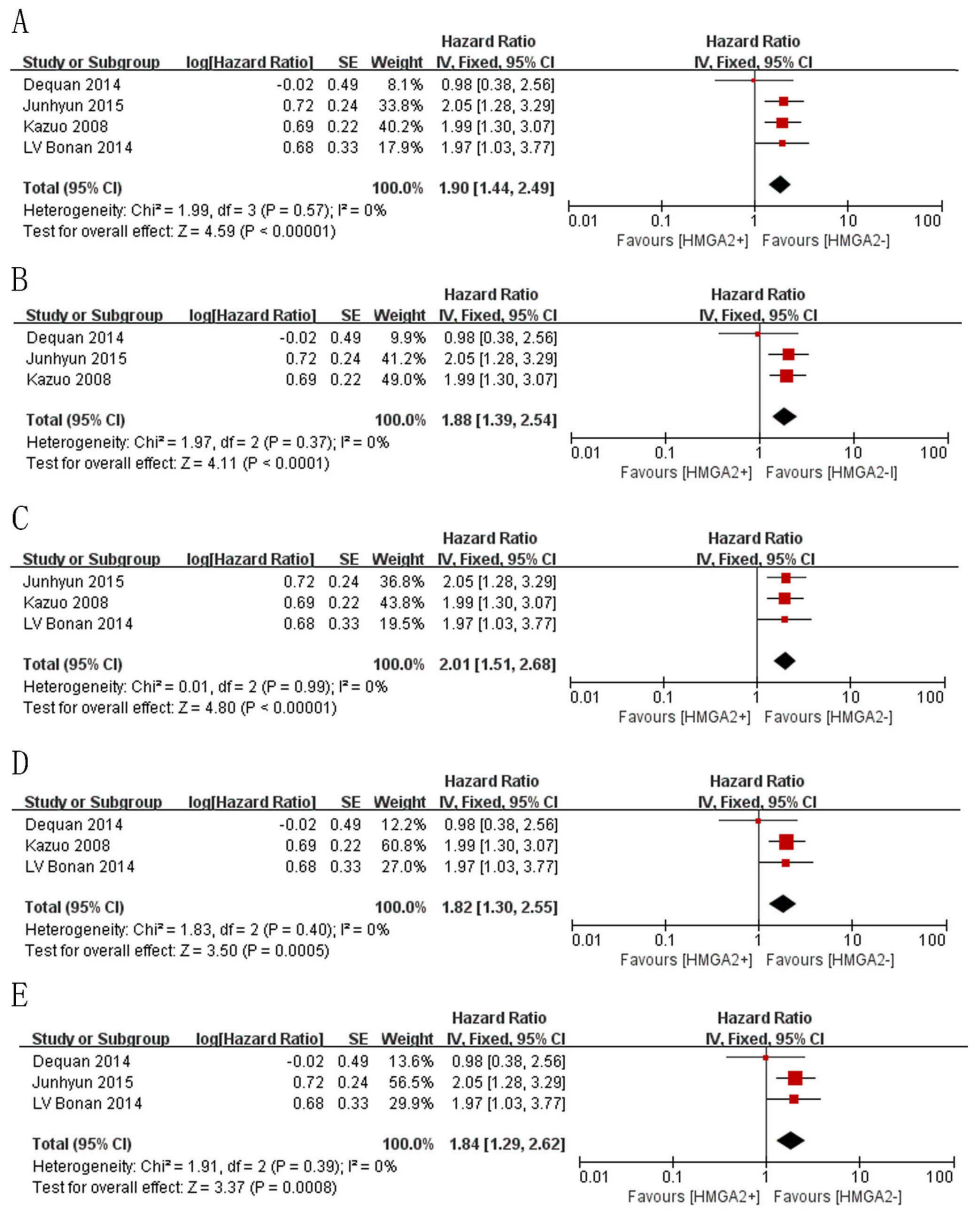
Sensitivity analysis estimating the impact of individual studies on the results of the meta-analysis of OS via sequential study removal **A**. Results when all studies were included. **B**. Results after the study conducted by LV Bonan et al. (2014) was removed. **C**. Results after the study conducted by Dequan et al. (2014) was removed. **D**. Results after the study conducted by Junhy et al. (2015) was removed. **E**. Results after the study conducted by Kazuo et al. (2008) was removed..

As depicted in Figure 5, the funnel plot indicated no evidence of publication bias in the reporting of the associations between clinicopathological features and OS. However, due to the limited number of studies included, it was difficult to confirm the absence of publication bias in this meta-analysis. In the sensitivity analysis, we sequentially omitted each study while repeating the analysis to assess the impact of individual studies on the pooled HRs calculated for OS. As shown in Figure 6, the results of the sensitivity analysis indicated that our meta-analysis of OS was not dominated by any single study; therefore, the conclusions herein demonstrated credibility.

## DISCUSSION

HMGA proteins are non-histone, architectural chromatin proteins that have been found to have prognostic value in lung cancer [2], oral squamous cell carcinoma [44, 45], ovarian cancer [8], breast cancer [46], and colorectal cancer [47] in terms of survival and recurrence rates. However, no previous studies have evaluated the prognostic value of HMGA2 overexpression in GC patients, which was the subject of our investigation.

The results of the overall analysis revealed that HMGA2 overexpression predicted poor prognosis in GC patients. Remarkable positive associations were identified between HMGA2 expression and clinicopathological characteristics, including lymph node metastasis, lymphatic invasion, TNM stage and vascular invasion, while insignificant associations were identified between HMGA2 and histological differentiation and sex. These results implied that HMGA2 may affect tumor progression but not tumorigenesis, as histological differentiation, which indicates very nature of a tumor, was not associated with HMGA2 overexpression. The OS of patients with high HMGA2 expression was poorer than that of patients with low HMGA2 expression. The results of the heterogeneity test showed that our results were stable for all variables except lymph node metastasis. Taken in conjunction, these data suggest that HMGA2 may help to determine prognosis in GC patients. In addition, our results suggest that HMGA2 was more likely to participate in tumor invasion and metastasis than tumorigenesis. Further studies are needed to support this conclusion.

Based on our previous study, HMGA2 promotes invasiveness and cell proliferation in GC by eliciting epithelial-mesenchymal transitions (EMT) and acquiring tumor stem cell properties through the activation of the HMGA2-TWIST1, HMGA2-FOXL2-ITGA2 and Wnt/β-catenin pathways, ultimately resulting in chemoresistance and distant metastases [48-51]. The inhibition of HMGA2 by Raf kinase inhibitor protein or miR-495 can suppress GC cell survival and invasion [52, 53]. Additionally, HMGA1, another member of the HMGA family that shares a similar structure with HMGA2, has also been recognized as an oncogene and may be overexpressed in many human malignant tumors[54-58] including GC[5, 59]. However, even though HMGA1 has been observed to be involved in cell proliferation and tumorigenesis, its efficacy as a prognostic indicator in GC remains under debate [40, 60, 61]. The results of the study conducted by Kyong-Hwa Jun [5] suggested that high HMGA1 expression levels were not necessarily associated with the clinicopathological features of malignancies, but opposing conclusions were derived in the study conducted by Nam ES [59]. Therefore, further mechanistic studies and large-scale experiments are necessary to ascertain whether the level of HMGA1 expression is associated with outcomes in patients with GC.

This study had several limitations that should be acknowledged, and some results need to be interpreted cautiously. First, the criteria adopted by included studies for defining HMGA2 positive or negative are different from each other. Three studies (Junhy 2015, Kyong-Hwa 2015, LV Bonan 2014) defined the positive and negative by multiplying the scores of expressing intensity and area. Two studies (Dequan 2014, ZHA Lang 2011) defined the positive and negative by the scores of expressing area only. And one study defined the positive and negative by the median HMGA2 mRNA expression level. Although we admit there are some differences between HMGA2 1+ and HMGA2 3+ in the included studies. However, we cannot discuss the differences due to the lack of original data record from enrolled studies. We hope there will be more studies assessing the relationship between different rankings and the prognosis in the future. Second, the source of antibody, concentration and evaluation method used in different included studies are different. we add a list of the source of antibody, concentration and evaluation method used in included studies in the supplement table, which are all diverse from each other. In this condition, making a subgroup analysis to evaluate the heterogeneity is not practical. It’s truly a significant source of heterogeneity, also an inevitable one. Third, all of the research institutes at which the included studies were performed were located in Asia, which suggests that the tissue samples were probably all obtained from Asian patients. Furthermore, the effects of some factors, such as age, gender and smoking habits, were not considered in this analysis because of insufficient data. Lastly, the majority of the included studies did not use blinding, which might have resulted in a selection bias.

Despite these limitations, this is the first meta-analysis to comprehensively evaluate the association between increased HMGA2 expression and prognosis in GC. The results of the heterogeneity tests give our conclusions credibility. We believe that HMGA2 has the following clinical significance as a prognostic biomarker: (1) this oncogene may help in the determination of clinical outcomes in patients with GC and provide early indications of the possibility of cancer recurrence or metastasis; (2) GC therapies involving HMGA2 gene suppression or silencing may be utilized; (3) this oncogene may help in the identification of high-risk patients who are good candidates for individualized treatment [3]; (4) selective use of chemotherapy agents, such as doxorubicin and cisplatin, may be employed if HMGA2 expression is found to correlated with chemoresistance; and (5) this oncogene may be used to help monitor responses to therapy and facilitate decisions regarding further treatment.

GC is a leading cause of human cancer mortality[42] that is predominantly induced by tumor invasion, metastasis, and recurrence. Current prognostic indicators have been unable to satisfy the need for accurate prediction of long-term outcomes in patients with GC. Therefore, our identification of HMGA2 as a biomarker in GC is of great value in the effort to provide relatively precise prognoses. Large-scale and prospective cohort studies will ultimately be needed to confirm the results of our study, especially in non-Asian countries. Moreover, further efforts are required to elucidate the mechanism underlying the involvement of HMGA2 in the progression of GC. Since multimarkers may provide more precise prognostic information, studies estimating HMGA2 overexpression in combination with other prognosis markers are also essential in the effort to assess the value of these indicators in GC survival.

## MATERIALS AND METHODS

### Literature search

The PubMed, Embase, Cochran Library, and CBM databases were searched to identify articles published from inception to June 1, 2016. The search strategy was as follows: (“HMGA2” or “HMGI-C”) AND (“gastric cancer” or“gastric carcinoma” or “stomach cancer” or “cancer of the stomach” or GC). The literature was searched, and relevant studies were independently selected by two authors. After excluding obviously irrelevant articles by screening titles and abstracts, full texts were obtained and reviewed as to whether the literature met the inclusion criteria.

### Inclusion criteria

The inclusion criteria were as follows: (a) cohort studies; (b) GC was evaluated; (c) HMGA2 expression was evaluated by immunohistochemistry (IHC) assay; (d) associations between HMGA2 expression and clinicopathological parameters and/or survival duration were assessed; and (e) cancerous tissues had been obtained from humans.

### Data extraction

Data were independently extracted from each eligible study by two investigators. When the extracted data differed between the two investigators, these differences were discussed until consensus was reached. The following data were extracted: basic study information, patient characteristics, HMGA2 detection method, HMGA2 positive ratio, and clinicopathological parameters.

### Methodological quality assessment

Two investigators independently evaluated the methodological quality of the included studies using the Newcastle-Ottawa Scale (NOS) [38]. Each study was scored based on the following three factors and available points: (1) selection, 0-4; (2) comparability, 0-2; and (3) outcome: 0-3. The full score was 9 points, and a score ≥7 indicated good quality.

### Statistical analysis

To estimate the impact of HMGA2 on overall survival (OS), we calculated HRs and their corresponding 95% CIs based on values that were directly reported in the literature or calculated using provided event data and the p value reported for the log-rank test; alternatively, HRs and variances were estimated based on overall survival curves when studies did not provide HR data [38]. To assess the associations between high HMAG2 expression and the evaluated clinicopathological parameters, we calculated relative risks (RRs) and their corresponding 95% CIs.

We determined the presence of statistically significant heterogeneity across studies using the chi-square-based Q test and the I^2^ test. If the observed heterogeneity was not significant (I^2^>50% or *p* < 0.10), a random-effects model was selected for summary estimation. Otherwise, a fixed-effects model was adopted.

A sensitivity analysis was performed to estimate the impact of individuals studies on the meta-analysis results for OS by sequentially removing each study. The presence of publication bias was evaluated using funnel plots.

All analyses were performed using Review Manager version 5.3 [62].

## SUPPLEMENTARY MATERIALS TABLE




