# Supplementary material for: Clinicopathological and prognostic significance of HMGA2 overexpression in gastric cancer: a meta-analysis
Source: Oncotarget. 2017 Jul 5;8(59):100478–89. doi: 10.18632/oncotarget.19001 (PMC5725036; doi:10.18632/oncotarget.19001)
Supplement: Supplementary file 1 [file oncotarget-08-100478-s001.pdf]

Clinicopathological and prognostic significance of HMGA2 overexpression in gastric cancer: a meta-analysis

Supplementary Material

For Supplementary Table see in Supplementray Files
