# Supplementary material for: Clinicopathological and prognostic significance of HMGA2 overexpression in gastric cancer: a meta-analysis
Source: Oncotarget. 2017 Jul 5;8(59):100478–89. doi: 10.18632/oncotarget.19001 (PMC5725036; doi:10.18632/oncotarget.19001)
Supplement: Supplementary file 2 [file oncotarget-08-100478-s002.docx]

**Supplementary Table 1: Concentration and evaluation method used in included studies**

| Study | Antibody | Concent-  ration | Evaluation method of HMGA2 expression level |
| --- | --- | --- | --- |
| Dequan et al. (2014) | rabbit primary antibodies against human HMGA2 (1:100; Cell Signaling Technology, Danvers, MA, USA) | NA | The expressions of HMGA2 were analyzed and scored as previously published using a semiquantitative score: score 0, no positive cells; score 1, positive cells<10 %; score 2, positive cells >=10 %; and score 3, positive cells>=50 %. Patients were subsequently categorized into either a positive expression group (score 1–3) or a negative expression group (score 0). |
| Junhy et al. (2015) | rabbit polyclonal anti-HMGA2 antibody (1:100; Abcam, Cambridge, MA, USA) | NA | The intensity of positive staining was scored as follows: 0, negative; 1, weak; 2, moderate; and 3, strong, and the extent of positive staining according to the percentage of positive cells in the respective lesions: 0, 0%; 1, 1-10%; 2, 11-25%; 3, 26-50%; 4, 51-75%; 5, 76-90%; and 6, >90%. The final score was obtained by multiplying the positivity and intensity scores, yielding a range from 0 to 18. HMGA2 expression was considered positive when the final score was ≥9. |
| Kazuo et al. (2008) | polyclonal antibody against human HMGA2 | 10 ug/mL | Based upon the median HMGA2 mRNA expression level, the 110 clinical cases were divided into two groups: high HMGA2 expression (n = 55) and low HMGA2 expression (n = 55). |
| Kyong-Hwa et al. (2015) | anti-HMGA2 antibodies (1:100; ab52039; Abcam Cambridge, MA, USA) | 2 ug/mL | For HMGA assessment, staining intensity was scored as 0 (lack of staining), 1 (weak staining intensity compared with control slide), or 2 (equal staining intensity compared with control slide). The extent of staining was scored as 0 (0%), 1 (1–25%), 2 (26–50%), 3 (51–75%), or 4 (76–100%) according to the percentages of the positive staining areas in relation to the whole carcinoma area. The final score was obtained semiquantitatively by multiplying the positivity and intensity scores, yielding a range from 0 to 8. For statistical analysis, the final staining scores of 0–4 and 5–8 were, considered low and high expression, respectively. |
| LV Bonan et al. (2014) | Rabbit/mouse polyclonal anti-human HMGA2 antibody (SANTA CRUZ, USA) | NA | For HMGA assessment, staining intensity was scored as 0 (lack of staining), 1(faint yellow), 2(pale brown), or 3(Medium Brown). The extent of staining was scored as 0 (0%), 1 (1–25%), 2 (26–50%), 3 (51–75%), or 4 (76–100%) according to the percentages of the positive staining cell at ten random high magnification vision. The final score was obtained by plusing the positivity and intensity scores, yielding a range from 0 to 8. For statistical analysis, the final staining scores of 0–3 and 4–8 were, considered a negative and positive expression group respectively. |
| ZHA Lang et al. (2011) | HMGA2 antibody (bioss, Beijing, China) | NA | The expressions of HMGA2 were analyzed as previously published using a semiquantitative method: a positive expression group: positive cells>=10%, a negative expression group: positive cells<10%. |
